# Therapeutic Potential of SphK1 Inhibitors Based on Abnormal Expression of SphK1 in Inflammatory Immune Related-Diseases

**DOI:** 10.3389/fphar.2021.733387

**Published:** 2021-10-19

**Authors:** Yanhong Bu, Hong Wu, Ran Deng, Yan Wang

**Affiliations:** ^1^ Key Laboratory of Xin’an Medicine, Ministry of Education, Hefei, China; ^2^ College of Pharmacy, Anhui University of Chinese Medicine, Hefei, China; ^3^ Anhui Province Key Laboratory of Chinese Medicinal Formula, Hefei, China; ^4^ Anhui Province Key Laboratory of Research and Development of Chinese Medicine, Hefei, China

**Keywords:** sphingosine kinase 1, inflammatory immune related-diseases, SPHK1 inhibitors, inflammatory immune response, disease

## Abstract

Sphingosine kinase 1(SphK1) a key enzyme that catalyzes the conversion of sphingosine (Sph) to sphingosine 1-phosphate (S1P), so as to maintain the dynamic balance of sphingolipid-rheostat in cells and participate in cell growth and death, proliferation and migration, vasoconstriction and remodeling, inflammation and metabolism. The normal expression of SphK1 maintains the balance of physiological and pathological states, which is reflected in the regulation of inflammatory factor secretion, immune response in traditional immune cells and non-traditional immune cells, and complex signal transduction. However, abnormal SphK1 expression and activity are found in various inflammatory and immune related-diseases, such as hypertension, atherosclerosis, Alzheimer’s disease, inflammatory bowel disease and rheumatoid arthritis. In view of the therapeutic potential of regulating SphK1 and its signal, the current research is aimed at SphK1 inhibitors, such as SphK1 selective inhibitors and dual SphK1/2 inhibitor, and other compounds with inhibitory potency. This review explores the regulatory role of over-expressed SphK1 in inflammatory and immune related-diseases, and investigate the latest progress of SphK1 inhibitors and the improvement of disease or pathological state.

## Introduction

Inflammatory immune response is a physiological or excessive response in cells, tissues or organs stimulated by changes *in vivo* and/or *in vitro*. Physiological inflammatory response is a beneficial defensive local response, but excessive inflammatory response will destroy the relative homeostasis of cells, tissues or organs, which is the pathological basis of the occurrence and development of a variety of diseases (McComb et al., 2019). Clinically, inflammatory response are often manifested as rubor, swelling, heat, pain and dysfunction. Their mechanisms are summarized as redness and swelling of tissues caused by congestion and edema in inflammatory lesions; fever and pain caused by inflammatory mediators such as interleukin-1 (IL-1) and prostaglandins (PG); dysfunction caused by degeneration and necrosis of intralesional parenchymal cells (Medzhitov, 2008). Therefore, inflammatory immune related-diseases are closely related to inflammatory mediators and inflammatory immune responses caused by immune cells, such as innate immunity involving phagocytes and dendritic cells (DCs), adaptive immunity involving lymphocytes and neutrophils, and inflammatory responses mediated by various inflammatory mediators. Inflammation and immune response are involved in a variety of diseases and even directly induces the occurrence of diseases, such as activation of the immune system and vascular inflammation in cardiovascular diseases, neuro-inflammation in Alzheimer’s disease (AD), and the well-known autoimmune disease-rheumatoid arthritis (RA) (Sorokin et al., 2020; Weyand and Goronzy, 2021). Therefore, the search for key inflammatory mediators and pro-inflammatory mechanisms has become a therapeutic target and strategy of various systemic diseases. In recent years, sphingosine kinase 1 (SphK1) has been recognized as a key factor in regulating inflammatory responses. The SphK1/S1P signaling mediates many normal and pathogenic inflammatory responses, including cytokines signaling mechanism and immune cell function regulation.

SphK1 is a key rate limiting enzyme and intracellular signal transduction enzyme in sphingolipid metabolism, which was first identified by Olivera in 1998 (Liu et al., 2000). As a lipid kinase, SphK1 phosphorylates sphingosine (Sph) to bioactive sphingosine-1-phosphate (S1P). S1P activates intracellular signals by binding to S1P receptors (S1PRs), members of the G protein-coupled receptors (GPCRs). SphK1/S1P/S1PRs signaling is a key regulator of several physiological processes, including Ca^2+^ homeostasis, cell survival, migration, and inflammation (Pyne and Pyne, 2020; McGinley and Cohen, 2021). Under normal physiological conditions, SphK1 located in the cytoplasm is transported to the plasma membrane and activated by a variety of stimuli, such as cytokines, growth factors and mitogen-activated protein kinase (MAPK), which participate in the enzymatic reaction and maintain the dynamic balance of intracellular sphingolipid metabolites. Under inflammatory conditions, abnormally expressed SphK1 and its product S1P participate in the regulation of inflammatory response and the functional management of multiple immune cells. SphK1 is abnormally activated by inflammatory stimulation, including endotoxin, tumor necrosis factor-α (TNF-α), interferon-γ (IFN-γ), IgE, etc., and the balance of its activity and expression is also disrupted (Obinata and Hla, 2019; Schneider, 2020). SphK1 plays a key role in the regulation of inflammation in metabolic syndrome (MS) and RA by triggering pro-inflammatory signals, regulating mitogenic and chemotactic responses of immune cells and the activation of vascular endothelial cells (VECs) (Chen et al., 2016; Wang et al., 2021). In addition, SphK1 was shown to be one of the earliest activated genes in IgE mediated mast cell initiation, participating in the survival and migration of immune cells (mononuclear phagocytes and lymphocytes), and contributing to the recruitment of immune cells to target tissues (Olivera et al., 2006). After SphK1/S1P pathway is activated, pro-inflammatory factors and various pro-inflammatory pathways are activated, including reactive oxygen species (ROS) and Toll-like receptors (TLR)signals, which have been proved in a variety of inflammatory immune related-diseases (Takasaki et al., 2018; Mohammed et al., 2020).

With the important role of SphK1 in autoimmune diseases, neurological and cardiovascular diseases, and cancer gradually revealed, the design and development of SphK1 targeted drugs has become a research hotspot at home and abroad. Most studies focused on the development of SphK1 selective inhibitors, based on the over-expression of SphK1 in inflammatory immune related-diseases and tumor pathology. At present, PF-543 is the most widely used SphK1 inhibitor, which participates in different cell functions to improve diseases. For example, PF-543 reduces mitochondrial DNA (mtDNA) damage in pulmonary epithelial cells, recruitment of fibrogenic monocytes, and ROS production to improve pulmonary fibrosis and lung injury (Cheresh et al., 2020; Ha et al., 2020; Huang et al., 2020). Studies on breast cancer and inflammatory bowel disease (IBD), including gastrointestinal tumors, showed that PF-543 reduces the expression of various immunosuppressive factors in the tumor microenvironment to overcome the resistance to immune checkpoint inhibitors (Imbert et al., 2020; Sukocheva et al., 2020a). In addition, it has been proved that more SphK1 inhibitors have been developed based on existing inhibitor structures or obtained from natural products. For example, jasmine B analogues based on pyrrolidine exerting anti-inflammatory activity for the treatment of RA (Chen et al., 2021a); therapeutic effect of SKI-349 obtained by optimizing SKI-178 structure on hematological malignancies (Hengst et al., 2020b); and the inhibitors derived form natural products with potential roles in the prevention of breast and lung cancer (Jairajpuri et al., 2020; Khan et al., 2020; Roy et al., 2020). More compounds with inhibitory activity have been developed and researched due to the benefits of SphK1 inhibitors have been proved. However, the selective screening, toxicity identification and cell function verification of these compounds need to be proved by more rigorous and careful studies. This review focuses on the association of SphK1 abnormalities with inflammatory immune related-diseases and pathological conditions, in order to clarify the importance for maintaining the homeostasis of SphK1 in inflammatory immune related-diseases. In addition, the recent research progress of SphKs inhibitors, including structure, cellular functions and possible molecular mechanisms.

## Sphingosine Kinase 1 and the Balance of Sphingolipid-Rheostat

**FIGURE 1:**
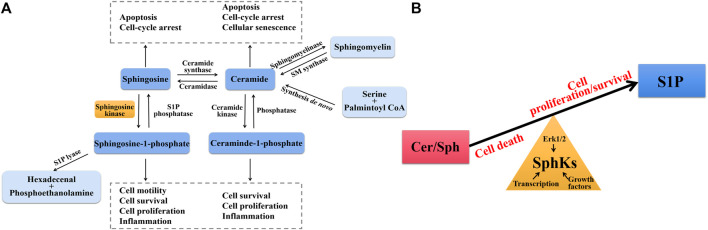
**(A)** Sphingolipid biosynthesis and degradation pathways. Ceramide (Cer) is the central core of sphingolipid metabolism, which is generated through the *de novo* synthesis pathway starting from serine and palmintoyl CoA, the SMase pathway by direct degradation of sphingomyelin (SM), and phosphorylation degradation of ceraminde-1-phosphate (C1P). Under the action of ceramidase, Cer is converted into sphingosine (Sph), which can be further phosphorylated by SphKs to form S1P. S1P lyase catalyzes irreversible exit from this pathway. Cer and Sph induce cell cycle arrest and apoptosis, whereas C1P and S1P promote cell proliferation and growth and induce inflammation. **(B)** The balance of sphingolipid-rheostat. Interconversion between Cer/Sph and S1P via SphKs. Cer/Sph induced cell death, whereas S1P promoted cell proliferation and survival. Sphingolipid-rheostat is formed between Cer/Sph and S1P, which determines cell death or survival. SphKs has intrinsic catalytic activity that helps maintain normal physiological levels through the balance of sphingolipid-rheostat, and is also influenced by Erk1/2, growth factors, and transcription level.

Sphingolipids are a complex class of lipids that were originally described as the main components of cell membrane structure. A core of sphingolipid metabolites is ceramide (Cer), which is converted to Sph after decarboxylation by ceramide synthase, and SphKs catalyzes Sph phosphorylation to produce S1P after activation by different cytokines and growth factors, such as TNF-α, IL-1β and vascular endothelial growth factor (VEGF) (Gomez-Larrauri et al., 2020). S1P is irreversibly degraded by S1P lyase (SPL) and S1P phosphatases (SPP) to maintain the dynamic balance of S1P in the body (Figure 1A). All of these sphingolipid metabolites have biological activities to regulate biological functions, including cell growth and survival, cell differentiation, autophagy, migration, and so on (Albeituni and Stiban, 2019). Among them, SphKs are rate limiting enzymes in maintaining S1P levels for cell survival and normal cell proliferation and function, determining cell fate by the balance between pro-apoptotic Cer/Sph and pro-survival S1P, which is called “sphingolipid-rheostat” (Newton et al., 2015). SphKs maintain the dynamic balance of sphingolipid-rheostat to reach relative homeostasis under normal physiological conditions. Whereas under pathological conditions, the abnormal expression of SphKs (especially SphK1) leads to disrupted homeostasis and altered biological functions of cells. This homeostatic environment is not only regulated by SphKs, the expression and activity of SphKs themselves are also influenced by the homeostatic environment. The activation of SphKs is mediated by three known ways, firstly by phosphorylation of the SphK1 Ser225 site by extracellular regulated protein kinases 1/2 (ERK 1/2), and secondly by external stimuli, especially growth factors and pro-inflammatory factors such as platelet derived growth factor (PDGF), TNF-α, transforming growth factor-β (TGF-β), in addition the activity of SphKs also be increased by up-regulating transcription level (Kono et al., 2010; Bonica et al., 2020). When the intracellular and external environments are disrupted by disease occurrence, SphKs are aberrantly activated leading to a disrupted balance of sphingolipid-rheostat (Figure 1B).

SphK1 and SphK2 are two isoforms of SphKs in mammals with subtle differences in substrate specificity and subcellular localization. SphK1 is mainly located in the cytoplasm, translocates to the plasma membrane when activated, and generates S1P to be transported extracellularly to exert biological effects through S1PRs (Wang et al., 2014a). SphK2 is located in various organelles, such as endoplasmic reticulum, mitochondria and nucleus, which are involved in apoptosis regulation and epigenetic regulation, respectively (Diaz Escarcega et al., 2021). The different localization of SphK1 and SphK2 determines the characteristics of mediating a variety of biological functions, such as the deposition of amyloid β-protein (Aβ) in AD and the pathogenesis of different cancers (Hatoum et al., 2017; Dominguez et al., 2018). In addition, the substrate specificity and structural differences of the two isoforms provide a basis for the development of targeted inhibitors. Therefore, we will focus on the role of abnormal SphK1 in inflammatory immune related-diseases, in view of the universality and importance of abnormal SphK1 expression and activity in the regulation of pathological state and biological function.

## Role of Sphingosine Kinase 1 in Inflammatory Immune Related-Diseases

**FIGURE 2:**
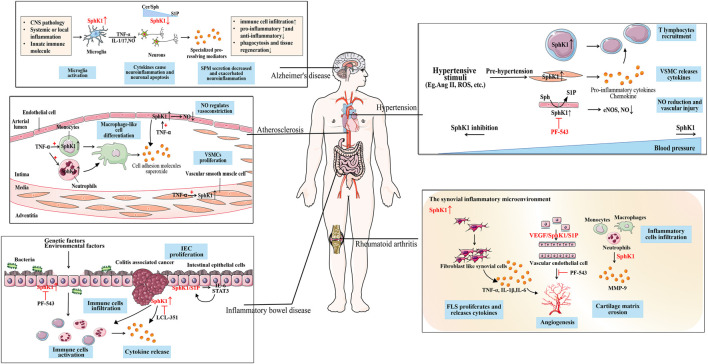
The role of SphK1 in inflammation immune-related diseases. SphK1 is over-expressed in a variety of inflammation immune-related diseases. Hypertension caused by the production of cytokines and various stimuli is accompanied by the activation of the immune system. In the inflammatory environment, the up-regulated SphK1 is involved in the recruitment of T lymphocytes to the inflammatory site, the release of inflammatory cytokines, and the vascular injury caused by the reduction of NO release in the pathogenesis of hypertension. As SphK1 is abnormally activated, SphK1 inhibition (such as PF-543) can effectively reduce blood pressure. Atherosclerosis is accompanied by the activation of the immune system. SphK1 activated by TNF-α promotes the differentiation of macrophage-like cells in immune cells, produces cell adhesion molecules and superoxide, and the proliferation of VSMCs. SphK1 over-expressed in ECs regulates the release of NO for vasoconstriction, which is the cause of vascular inflammation. SphK1 is over-expressed in activated microglia, promotes the release of pro-inflammatory mediators, and causes neuroinflammation and neuronal apoptosis in AD. Whereas SPM reduction is associated with down-regulated SphK1 in neurons, aggravating neuroinflammation which may be due to sphingolipid-rheostat modulation. SphK1 over-expression activates immune cells to release cytokines in the IEC of IBD caused by genetic and environmental factors. When developing into CAC, SphK1 induces the expression of pro-inflammatory markers and immune cell infiltration, which can be alleviated by SphK1 inhibition (such as LCL351). SphK1/S1P forms a positive feedback with downstream IL-6/STAT3 to maintain the proliferation of IEC. SphK1 is abnormally over-expressed in the synovitis microenvironment of RA, participates in FLS proliferation, the release of inflammatory cytokines, and inflammatory cell infiltration. Over-expression of SphK1 mediates the proliferation of ECs and forms neovascularization, which may be related to VEGF/SphK1/S1P signal. SphK1 inhibition (such as PF-543) can improve synovial microvascular angiogenesis.

Inflammatory immune response is a physiological or excessive systemic response induced by inflammatory immune cells based on the changes in the internal and external environments (Wei, 2016). Inflammation immune related-diseases are systemic diseases that involve excessive immune cells and inflammatory mediators, characterized by the occurrence of inflammatory immune responses as the basis of pathogenesis. Inflammatory immune related-diseases involve all aspects of the body and are classified as cardiovascular diseases, nervous system diseases, digestive system diseases and immune system diseases according to the type of disease. In addition to immune cells that play a central role in the process of immune response, such as neutrophils, monocytes, macrophages, T and B lymphocytes, there are non-traditional inflammatory cells involved in immune effects, including fibroblasts and endothelial cells. They release inflammatory cytokines, such as TNF family, ILs, chemokines and adhesion molecules, which mediate cell activities and regulate inflammation (Gajewski et al., 2013). SphK1 is highly expressed in various immune cells and tumor cells, and enhances the inflammatory response by triggering pro-inflammatory signals (Pyne and Pyne, 2020). As a key regulator regulating inflammation and immune responses, new potential targets have been found for the treatment of inflammation related diseases. Safingol, an SphK1 inhibitor, has entered the clinical stage for the treatment of solid tumors and leukemia (Dickson et al., 2011). Abnormal SphK1 has also become the research direction of a variety of diseases due to the discovery of over-expression of SphK1 in cancer. In this context, we will focus on the functional impact of altered SphK1 levels in a number of related cardiovascular, neurological disorders to explore novel drug targets for the treatment of inflammatory immune related-diseases (Figure 2).

### Cardiovascular Disease: Hypertension and Atherosclerosis

Hypertension caused by increased peripheral vascular resistance is the main risk factor of cardiovascular disease (CVD) in the world. Blood pressure has been focused on as a major CVD symptom for many years, but inflammatory processes and the immune response (including innate and adaptive immune responses) have been proved to play an important role in the pathogenesis of hypertension, participating in the elevation of blood pressure and end organ damage. Infiltration of immune cells (monocytes/macrophages, T lymphocytes) in perivascular fat, kidney and myocardium, increased expression of adhesion molecules and chemokines, and ROS generation are consistent features of hypertension (Prat et al., 2021; Zhang et al., 2021).

It has been proved that SphK1 is involved in the occurrence of pulmonary arterial hypertension (PAH) based on the over-expression of SphK1 in vascular smooth muscle cells (VSMC) and lymphocytes of patients with PAH, and the symptoms of PAH were alleviated in SphK1 knockout mice rather than SphK2 (Pyne and Pyne, 2017; Yang et al., 2019). Thus, SphK1/S1P signaling is a novel pathway in the inflammatory mechanism of PAH. Over-expression of SphK1 activates SphK1/S1P signaling to regulate S1P production, resulting in a concentration gradient change of S1P between tissues and blood (high in tissues and low in circulation), which promotes T cells to be excreted from lymphoid tissues and recruited to inflammation sites under the regulation of S1PR1 expressed on T cells. The S1PRs modulator FTY-720 has been shown to be effective in treating experimental hypertension by inhibiting S1PR1 mediated T cell outflow (Don-Doncow et al., 2019). In addition, SphK1 over-expression in VSMC increased IL-6 and signal transducer and activator of transcription (STAT)1 gene expression and up-regulated the expression levels of pro-inflammatory factors IL-8/33 and chemokines CXCL-6/8, as was found in PAH patients (Hirafuji et al., 2002; Bai et al., 2021). SphK1 not only participates in the inflammatory process in hypertension, but also plays a regulatory role in the process of vascular damage. Inhibition of SphK1 has therapeutic significance because of the high residual risk of hypertension caused by vascular damage and cardiac dysfunction. NO synthesized by endothelial cells acts as a vasodilator resists vasoconstriction induced by the sympathetic nervous system, and regulates the function and survival of T cells as a component of innate immunity, playing an important role in vascular damage and immune system in hypertension (Hu et al., 2020). PF-543 reduced inhibitory eNOS phosphorylation at T495 site and improved endothelial function in arteries from hypertensive mice. Although the mechanism is not clear, down-regulation of STAT3, protein kinase C (PKC) and ERK1/2 was observed in VECs (Józefczuk et al., 2020). Therefore, inhibition of SphK1 may be a promising target to solve the residual cardiovascular risk of hypertension.

The concept that atherosclerosis is an inflammatory disease is based on the finding that atherosclerotic lesions are rich in immune competent cells and produce large amounts of pro-inflammatory cytokines (Pedro-Botet et al., 2020). The accumulation of low density lipoprotein in arterial intima is the initial event of atherosclerosis. Accumulation of lipoproteins on the epithelium leads to local inflammation as well as expression of adhesion molecules, such as vascular cell adhesion protein 1 (VCAM-1). Monocytes are recruited and differentiated into macrophages under the action of granulocyte-macrophage colony-stimulating factor (GM-CSF), and VSMCs also differentiated into macrophage-like cells. Macrophages become the predominant cell population in atherosclerotic plaques due to continuous recruitment, differentiation, and local proliferation. DCs, mast cells, neutrophils and T cells are also present in the lesions, although not as commonly as macrophages (Wolf and Ley, 2019). TNF-α rapidly activates SphK1 in monocytes leading to the expression of adhesion factor, which has been shown to play a key role in the early stages of atherosclerosis pathogenesis (Zhi et al., 2006). Abnormally activated SphK1 has been shown to stimulate superoxide production in monocytes and neutrophils, which is inhibited by DHS to reduce inflammation (Vaidya et al., 2019). Macrophage apoptosis is considered to be a key step in the formation of unstable plaque in atherosclerotic. Macrophage apoptosis is inhibited by FTY-720 and PF-543 through cross-talk of macrophage polarization and autophagy and S1P signal, which helps to prevent the formation of unstable plaque (Sun et al., 2018; Zhu et al., 2020). Furthermore, activation of SphK1 is essential in vascular inflammation mediated by inflammatory cytokines such as TNF-α. Abnormal activation of SphK1 induced by TNF-α increased the expression of adhesion factor, leukocyte influx and VSMC proliferation. The survival, proliferation and migration of VCEs are regulated by SphK1/S1P signal, which is the cause of vascular inflammation (Chen et al., 2004).

### Neurological Disease: Alzheimer’s Disease

AD, a progressive and irreversible neurodegenerative disorder, which is the most common cause of dementia disease. The main pathological features of AD are the extracellular deposition of Aβ, neurofibrillary tangles (NFT) caused by hyperphosphorylated tau protein deposition, and extensive neuronal loss (Busche and Hyman, 2020). For a long time, neuroinflammation associated with AD has been considered to be only a response to pathophysiological events. However, new data from clinical studies have confirmed that inflammation and immune system mediated effects actually contribute to the pathogenesis of AD, including various immune cells, inflammatory cytokines and chemokines. Firstly, accumulated Aβ can activate immune related transcription factors (NF-κB) and produce inflammatory mediators (TNF-α, COX-2, iNOS) by binding to inflammatory receptors (TLR2, TLR4), indicating that Aβ is part of up-regulated physiological acute innate immunity (Newcombe et al., 2018). Secondly, neuronal inflammatory signals directly activate neuronal protein kinases and phosphatases, such as ERK and protein phosphatase 2A (PP2A), which regulate tau protein phosphorylation and neuronal microtubule assembly. Nervous system resident cells, such as microglia, eventually lead to cell functional damage and neuronal loss under this chronic inflammatory environment (Webers et al., 2020). The classical amyloid hypothesis and inflammatory mechanism drive the development of AD together.

The changes of sphingolipids and their metabolites in the brain, as well as their effects on neuronal homeostasis and immune system, provide a new strategy for understanding neurodegenerative diseases. The imbalance of Cer/S1P was observed from the earliest stage of clinically identifiable AD. The increase of pro-apoptotic Cer and the decrease of pro-survival S1P lead to the deposition of Aβ and apoptosis (Haughey et al., 2010). The cell fate and the relative level of these three mutually convertible sphingolipid metabolites are strongly affected by SphKs activity. There are different reports on the role of SphK1 in the pathogenesis of AD, especially in the production of Aβ. Aβ production was reduced in SphKs inhibitor treatment, SphKs knockdown or SPL over-expression. Here, the role of SphKs inhibitors is to reduce the production of S1P, prevent S1P specifically binding to full-length BACE1 and increase its proteolytic activity to reduce the production of amyloid (Takasugi et al., 2011). However, subsequent studies found that decreased SphK1 expression and increased of SPL expression in neurons were related to Aβ deposition in the AD brain. That is Aβ deposits are directly involved in the reduction of S1P by regulating the expression and activity of SphK1 and may ultimately alter the balance of death and survival, which is conducive to the neurodegenerative process (Ceccom et al., 2014). Consistent evidence is that SphKs inhibitors exacerbate cell death and increase Aβ deposition (Gassowska et al., 2014; Wang and Yang, 2021). Compared with the role of SphK1, dysregulation of SphK2 may have a more significant impact on AD lesions and progression. The increase (Takasugi et al., 2011) or decrease (Couttas et al., 2014) of SphK2 in different regions of AD brain lead to the controversial role of SphK2, but the intracellular localization of SphK2 seems to explain the complexity of regulation and function. The balance of SphK2 between cytoplasm and nucleus is destroyed in AD. Specifically, the decrease of pro-survival SphK2 in cytoplasm is negatively correlated with Aβ deposition, which promotes the pathogenesis of AD. The increased expression of SphK2 preferentially located in the nucleus of AD brain may be harmful to the pathogenesis of AD, which may be related to the destruction of the protective effect of retinoic acid (Dominguez et al., 2018).

In addition, as a natural immune cell of the central nervous system, microglia activation is considered to be one of the causes of neuroinflammation in various neurodegenerative diseases. SphK1 is over-expressed in microglia under inflammatory environment and promotes the expression and production of pro-inflammatory factors TNF-α, IL-1 and NO, which can be inhibited by DMS (Bryan et al., 2008; Lee et al., 2018). The increased expression of SphK1 also occurred in ischemia-induced neurons, and SphK1 inhibition attenuated neuroinflammation (Su et al., 2017). However, SphK1 negatively regulates the reduction of specialized pro-resolving mediators (SPM) and the aggravation of neuroinflammation (Lee et al., 2020). SPM has a strong role in promoting decomposition, resulting in the cessation of immune cell infiltration, down-regulation of pro-inflammatory mediators and up-regulation of anti-inflammatory mediators, as well as promoting phagocytosis and tissue regeneration (de Wit et al., 2020). These different findings make us realize that there is not only a positive effect on SphKs inhibition as a therapeutic strategy for AD.

In recent years, a large number of studies have confirmed that FTY-720 has shown therapeutic effect on nervous system diseases other than multiple sclerosis (Bascuñana et al., 2020). The widespread expression of S1PRs provides evidence for the beneficial effect of FTY-720, including neurons, astrocytes, microglia and immune cells. The regulation of FTY-720 on S1PRs not only inhibited the infiltration of immune cells, but also affected the synaptic function of astrocytes and microglia, thereby exerting anti-neuroinflammatory effects (Kartalou et al., 2020; Yin et al., 2021). In addition, FTY-720 also showed specific effects, such as down-regulating the expression of Cer to reduce Aβ production, so as to reduce a neurotoxicity and neuronal death (Jęśko et al., 2020).

### Digestive Disease: Inflammatory Bowel Disease

IBD is an intestinal immune inflammatory disease caused by intraluminal bacteria. Crohn’s disease and ulcerative colitis (UC) are two clinical manifestations of IBD characterized by chronic recurrent intestinal inflammation. Although the etiology of IBD remains largely unknown, it involves complex interactions between genetic, environmental or microbial factors and immune response (Czubkowski et al., 2020). The common feature of IBD immune response is that mast cells, monocytes, macrophages and neutrophils invade the colonic epithelial layer, and then amplify the inflammatory process. Dysfunctional mucosa promotes the infiltration of immune cells into the gastrointestinal tissue, and then various pro-inflammatory pathways are activated, including ROS, arachidonic acid, TLR and TNF-α signal (Wallace et al., 2014).

Sphingolipid signalling was found to be one of the key mediators in the pro-inflammatory mechanisms of IBD since over-expressed SphK1 and high concentrations of S1P in plasma, lymphocytes of patients with gastrointestinal cancer (Degagné and Saba, 2014; Sukocheva et al., 2020b). Increased SphK1 and S1PR1 were detected in the inflammatory mucosa of UC patients, and PF-543 inhibited the inflammatory response in UC mice. Notably, over-expression of SphK1 and S1P has been shown to promote proliferation of intestinal epithelial cells (IEC) by enhancing c-Myc expression, inducing pro-inflammatory cytokines secretion to promote monocyte macrophage proliferation via JAK2/STAT3 signaling (Abdin, 2013; Liu and Jiang, 2020). Moreover, the different cellular localization of SphK1 is critical for the downstream effects. Haematopoietic cell-derived SphK1 regulates circulating S1P concentrations, which in turn stimulate neutrophilia, splenic lymphocyte egress and systemic inflammation. SphK1 located in extrahematopoietic cells (such as IEC) is involved in the regulation of COX-2 expression mediated by TNF-α signaling (Furuya et al., 2017). IBD is a prominent example of the link between chronic inflammation and cancer, and one of the consequences of persistent inflammation is colitis associated cancer (CAC). Over-expressed SphK1 is more prevalent in patients with CAC (Yuza et al., 2018). The expression of pro-inflammatory markers of CAC (such as CXCL1/2) and S1P tissue concentration decreased after treatment with SphK1 selective inhibitor LCL351, preventing leukocyte recruitment and reduce neutrophil infiltration. In addition, SphK1 and S1P activated by STAT3 in IEC act as upstream mediators of pro-inflammatory cytokines IL-6 and STAT3, which in turn activate SphK1/S1P signal (Liang et al., 2013; Park et al., 2020). This axis maintains STAT3 activation in IEC through a positive feedback loop. Therefore, sphingolipid signaling, especially SphK1, may be a therapeutic target for IBD and CAC.

### Immune System Disease: Rheumatoid Arthritis

RA is a chronic, destructive and autoimmune disease characterized by excessive inflammation of joint tissue, chronic inflammatory infiltration of the synovium, and microvascular formation, which eventually leads to the destruction of bone and cartilage (Testa et al., 2021). The inflammatory environment of RA joint is composed of many kinds of cells, including fibroblast-like synovial cells (FLS) and VECs, as well as immune cells, such as T and B cells, macrophages and monocytes. The production of inflammatory cytokines by activated cells mediates the interaction between cells, such as TNF-α, IL-1β, IL-6 and so on (Yang et al., 2016).

The abnormally high expression of SphK1 has been found in the synovial fluid of RA patients, and S1P levels were also significantly higher than in patients with osteoarthritis (Lai et al., 2012). In addition, SphK1^−/−^ mice showed significantly reduced synovitis and joint pathology, and PF-543 and FTY-720 have also been shown to exert therapeutic effects against inflammation and angiogenesis in RA by regulating SphK1 and S1P/S1PRs signals, respectively (Bougault et al., 2017; Wang et al., 2021). S1P/S1PRs signals are involved in the biological function changes of RA, including FLS proliferation, migration, inflammatory cell infiltration, and induced COX-2 expression and PGE2 production (Baker et al., 2011). The dynamic balance between pro-/anti-angiogenic factors was disrupted in abnormally proliferating FLS and VECs, synovial microvascular neogenesis was induced by VEGF mediated SphK1/S1P/S1PR1. PF-543 exert an anti-angiogenic effect by inhibiting SphK1 (Sun et al., 2020; Deng et al., 2021). The inflammatory microenvironment of synovium is produced by a large number of inflammatory cells infiltration, which is characterized by up-regulated cytokines, such as TNF-α, IL-1β and IL-6 (Kuwabara et al., 2017). Matrix metalloproteinase-9 (MMP-9) release from macrophages and neutrophils is thought to play a key role in inflammatory cell migration and cartilage matrix erosion (Yuan et al., 2014), while SphK1 inhibition reduce the release of cytokines and MMP-9 in monocytes and alleviate synovitis. These results clearly indicate that the potential of SphK1 inhibition not only reduces synovial inflammation, but also improves immune cell infiltration and angiogenesis.

## Development of Sphingosine Kinase 1 Inhibitors

In recent years, SphK1 has been increasingly recognized as a major driver of various inflammatory diseases and cancers, and there is great interest in inhibiting over-expression of human SphK1 (hSphK1) as an anticancer therapy. However, the drug design of SphK1 inhibitors has inherent complexity due to the diversity of SphKs functions and its important role in normal physiology.

The hSphK1 gene is located in 17q25.2, and there are three major isoforms, SphK1a, SphK1b, and SphK1c, respectively. SphK1a is mainly involved in extracellular signal transduction, whereas SphK1b and SphK1c are anchored to the plasma membrane. SphK1 consists of N-terminal (NT) and C-terminal (CT) domains, including 9 a-helices, 17 b-strands, and 310 helices, whereas the catalytic domain is located in the gap between the two domains (Wang et al., 2013). The two-domain structure of SphK1 belongs to phosphofructokinase (PFK)-like superfamily and has no similarity with protein kinase or other lipid kinases (such as PI3K). Although SphK1 shares the same protein fold with DGKs, NAD kinases, which also belong to the PFK superfamily. However, lipid molecules deeply bind to the protein through the unique folding of lipid binding cavity CTD, conferring the substrate specificity of SphK1 (Garavaglia et al., 2004). In addition, the motifs contained in the five conserved domains have specific binding sites closely related to their functions, such as active sites, nucleotide binding sites, magnesium ion binding sites, calcium/calmodulin coupling sites, lipid binding sites, and phosphorylation/dephosphorylation sites (Wang et al., 2013). Among them, adenosine triphosphate (ATP)-binding motif allows the transfer of γ-phosphoryl from ATP to D-erythro sphingosine to produce S1P. It has been demonstrated that SphK1 binding sites occupy a J-shaped channel, whether substrate Sph or inhibitors (such as PF-543) (Li et al., 2021). The crystal structures of PF-543 and Amgen 82 which bind to SphK1, indicate that the aminoalcohol portion is oriented toward the ATP binding pocket and interacts with two key aspartate residues that are critical for enzyme activity via hydrogen bonds. The discovery of the crystal structure of SphK1 plays an important role in the development of SphKs inhibitors. Although the structure of SphK1 has been confirmed, the specific function and molecular mechanism, such as activation by translocation, or the interactions between different isoforms and other molecules, remain poorly understood.

There has been great interest in drugs targeting SphK1 as a potential target due to the importance of SphK1 in inflammatory immune related-diseases. SphK1 inhibitors have been studied extensively, from molecular structure to pharmacological effects. Next, we review SphK1 inhibitors, including selective SphK1 inhibitors, dual SphK1/2 inhibitors and other compounds with inhibitory activity.

## Sphingosine Kinase 1 Selective Inhibitors

### Sphingosine Analogues

#### SK1-I

**FIGURE 3:**
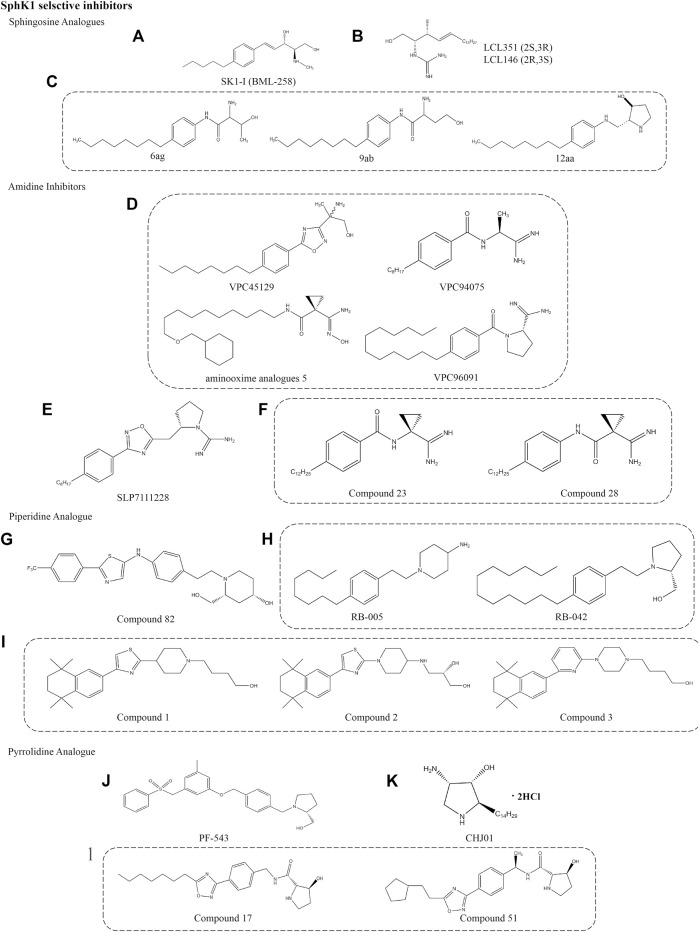
Structures of selective SphK1 inhibitors.

SK1-I (BML258), 1 (2R, 3S, 4E)-N-methyl-5-(4-pentylphenyl)-2-aminopent-4-ene-1, 3-diol, is sphingosine analogues discovered in 2008 by Paugh et al. (Figure 3A) (Paugh et al., 2008). SK1-I is a competitive and selective SphK1 inhibitor with Ki value of 10 μM (Pitman and Pitson, 2010), and has been widely used to elucidate the role of SphK1 in cancer. In the mouse model of breast cancer, SK1-I decreased serum S1P level, stimulated cancer cell apoptosis, and reduced angiogenesis and lymphangiogenesis (Nagahashi et al., 2012). SK1-I also reduced S1P levels and increased the expression of Cer derivatives in human leukemia U937 cells by inhibiting SphK1, which is related to the decrease of ERK1/2 and Akt signals (Paugh et al., 2008). Recent studies have shown that SK1-I increased the transcriptional activity of tumor suppressor protein TP53 and the expression of pro-apoptotic members of the downstream BCL2 family, thereby enhancing autophagy and cancer cell death (including colon cancer and breast cancer) in a SphK1 dependent manner (Lima et al., 2018). In addition, SK1-I negatively regulates the expression of MMPs and cell migration and invasion, which is related to the alleviation of various pregnancy related diseases (Chahar et al., 2021). Compared with other SphK1 inhibitors, SK1-I has high solubility and allowed its delivery *in vivo*, which makes it an ideal SphK1 inhibitor and used in a wide range of animal models. However, some studies have found that SK1-I plays a cytotoxic role in AML models (Paugh et al., 2008), suggesting that more research is needed for the cytotoxic effects to be applied in disease treatment.

#### LCL351/146

LCL351 and LCL146 are potent SphK1 inhibitors that are Sph analogues. Unlike the Sph structure, their polar head amino group is replaced by a guanidine group. The chemical names are L-erythro-2-N-(1’-carboxamidine)- sphingosine hydrochloride and D-erythro-2-N-(1’-carboxamidine)-sphingosine hydrochloride, respectively (Figure 3B). They act as two erythro diastereoisomers, showing different inhibitory potency due to different stereochemistry at C2 and C3 positions. LCL351 was found to be more potent than LCLl46, suggesting a stereochemical interaction with target cells. LCL351 was identified as a SphK1 selective inhibitor with Ki values of 5 and 50 μM against SphK1 and SphK2, respectively. *In vitro* studies showed that LCL351 induced SphK1 degradation and had no effect on cell death and cell cycle, thus it may not cause side effects (Sharma, 2011). Studies in a mouse model of IBD found that LCL351 reduced plasma S1P levels as well as the expression of pro-inflammatory cytokine, alleviated neutrophil infiltration and immune responses, and exerted therapeutic effects (Pulkoski-Gross et al., 2017).

#### 6ag/9ab/12aa

This novel class of SphK1 selective inhibitors was engineered on the basis of Sph to design a series of compounds that replace the aminodiol headpiece of Sph with a serine amide (Figure 3C). Such a structure not only increases the affinity of new molecules to SphK1, the carboxylic acid of serine also provides a convenient synthetic site to bind various mimetics for the lipophilic tail of Sph. The activity was significantly increased when L-threonine was used as the polar headpiece, and the resulting compound 6ag was nearly 10 times higher. To explore the effect of the distance between the terminal alcohol and the amide group, a series of compounds were prepared and found that the greater the distance, the lower the inhibitor potency. Moreover, the potent of S-enantiomer 9ab was approximately 40 times that of R-enantiomer (50 nM vs. 2.2 μM), indicating that the stereochemistry of homoserine analogues has a significant effect on activity. Compound 12aa is a modification of the polar headpiece with 3-hydroxyproline that is much more potent. In this series of inhibitors, the amide functionality was critical for inhibitor potency and significantly more potent than the previously reported SphK1 inhibitor N, N-dimethylsphingosine. Although no more studies on this series of compounds have been reported, 3-hydroxyproline provides more possibilities for the development of selective inhibitors to improve the activity and ADME properties *in vitro* (Xiang et al., 2009).

### Amidine Inhibitors

#### VPC96091

The design and biological activity evaluation of a series of amide SphKs inhibitors were reported by Frank W et al. (Figure 3D) (Foss et al., 2009). It is worth noting that VPC45129 is the first alcohol compound with significant activity on SphK1. This special compound containing alcohol is attractive because few synthetic analogues are active on SphK1. Compound VPC94075 has dual inhibitory effects on SphK1 and SphK2 with Ki values of 55 and 20 μM, respectively, and has the effects of reducing S1P level and anti-proliferation *in vitro*. Therefore, a series of amidine-based compounds were synthesized and optimized as SphKs inhibitors with VPC94075 as the leading compound.

VPC96091, an effective selective SphK1 inhibitor, was found to be further developed through the above inhibitors. It chemical name is (S)-1-(4-dodecylbenzoyl)pyrrolidine-2-carboximidamide hydrochloride, which is characterized by a terminal α-substituted amino group linked with 4-alkyl phenyl via amide bond. The Ki values for SphK1 and SphK2 were 0.10 and 1.50 μM, respectively. Selective inhibition of SphK1 by VPC96091 reduced epidermal growth factor (EGF) driven S1P levels and increased Akt/ERK phosphorylation in human leukemia U937 cells and mice model (Kharel et al., 2011). In the patent reported by the University of Virginia, a series of SphKs inhibitors with amide bonds are described, including aminooxime analogues 5. It is different from VPC96091 because the terminal α-substituted amino group is linked by a linear alkyl group (University of Virginia Patent Foundation, 2014). The conversion of amidines into amidoximes can improve the oral bioavailability of drugs according to the prodrug principle (Kotthaus et al., 2011).

#### SLP7111228

SLP7111228, an effective selective inhibitor of SphK1 with Ki value of 48 nM, which was obtained after modification of SphK2 inhibitor SLP120701. Its chemical name is (S)-2-((3-(4-octylphenyl)-1,2,4-oxadiazol-5-yl)methyl)pyrrolidine-1-carboximidamide hydrochloride and its structure is based on guanidine, which was published by Patwardhan et al. (Figure 3E) (Patwardhan et al., 2015). Studies have confirmed that SLP7111228 can reduce the level of S1P in U937 cells, and the same effect has been proved *in vivo* studies of mice and rats models. Another compound in the same series, SLP080801, is defined as a selective SphK2 inhibitor that improves S1P levels in the circulation of mice (Kharel et al., 2012). The inhibition or gene deletion of SphK2 is the key to the increased accumulation of S1P in the blood, but its mechanism is still unclear. This suggests that this increase may be regulated by the dynamic balance between SphK1 and SphK2, which are responsible for the synthesis and elimination of S1P in the blood in some way.

#### Compound 28

Compounds 23 and 28 are the most interesting of the amido derivatives, which are derived from VPC45129 step by step (Figure 3F). Mathews et al. found moderate inhibition of SphKs after catalytic ring-opening and re-arrangement. Then, the methyl group was replaced by cyclopropyl ring to increase the steric bulk of the junction region, and some cyclopropyl derivatives with different tail lengths were evaluated in order to determine the optimal tail length. It is worth noting that these compounds retain the unique chemical properties of amides, which is very important for inhibitory potencies. Finally, an effective dual SphK1/2 inhibitor (Compound 23) and an effective selective SphK1 inhibitor (Compound 28) were identified after evaluating the inhibitor potency, selectivity and *in vitro* activity of the inhibitors. The Ki values of compound 23 for SphK1 and SphK2 were 0.2 and 0.5 μM, respectively. Compound 28 showed good selectivity for SphK1, with Ki values of 0.3 μM for SphK1 and 6 μM for SphK2. Specificity evaluation did not find the inhibitory effect of the two compounds on diacylglycerol kinases (γ, δ1, ζ) and protein kinase C family, PKC α. The availability in smooth muscle cell-based disease models demonstrates the pharmacodynamic potential of these novel SphKs inhibitors. In addition, more studies have begun to identify pharmacophores related to aminosphingosine kinase inhibitors (Mathews et al., 2010).

### Piperidine Analogue

#### Compound 82

Compound 82 is a selective SphK1 inhibitor obtained by optimizing the structure of dual SphK1/2 inhibitor SKI-Ⅱ. It competitively inhibits SphK1 with IC50 of 0.02 and 0.10 μM for SphK1 and SphK2, respectively (Figure 3G) (Gustin et al., 2013). Hydrogen bonds are formed by the aminoalcohol portion of compound 82 and two key residues of Asp in SphK1, which play a key role in the interaction between the compound and the target molecule, just like PF-543 (Wang et al., 2013). Asp178 forms hydrogen bonds with nitrogen on the piperidine ring and hydroxyl outside the piperidine ring, respectively. Asp81 also forms hydrogen bonds with hydroxyl on the piperidine ring. In addition, *in vivo* and *in vitro* pharmacokinetic parameters showed good mean residence time (MRT) and the bioavailability of 32% (Gustin et al., 2013). Pharmacological studies have shown that compound 82 can reduce the level of S1P in human breast and melanoma cell lines, but has no effect on the growth of tumor cells (Rex et al., 2013). Unfortunately, these results have not been confirmed *in vivo* studies.

#### RB-005

RB-005, chemical name 1-(4-octylphenethyl)piperidin-4-amine, is a derivative obtained from the route of synthesizing FTY-720 from 4-octylphenylethanol (Figure 3H) (Baek et al., 2013a). RB-005 was identified as a selective SphK1 inhibitor based on Sph with IC50 of 3.6 µM. The compound has an n-octylphenyl group linked in a 2-carbon tether to the nitrogen of 4-hydroxypiperidine. This small change in tertiary amine structure is responsible for supporting that RB-005 maintains the selectivity for SphK1. The hydroxyl groups and size of heterocycles are also crucial for selectivity, such as RB-042, which is converted into a dual inhibitor of SphK1/2 with the IC50 of 2 µM for both isoforms. The specificity of RB-005 for SphK1 was established by a study that found that RB-005 induced SphK1 proteasome degradation in human pulmonary artery smooth muscle cells, which was reversed by proteasome inhibitor MG132 (Baek et al., 2013b). Other studies found that RB-005 inhibition of Cer synthase has an effect on lung and heart remodeling in the hypoxic model of pulmonary hypertension mice (MacRitchie et al., 2016). However, more evidence is needed to prove that the affinity for SphK2 and other bioactive enzymes.

#### Compound 1/2/3

These are a series of selective inhibitors of SphK1 based on the framework of 2-piperidine thiazole, which were published by Merck Serono (Figure 3I) (Frank, 2013). These compounds are usually occupied by a 5,5,8,8-tetramethyl tetralin (Compound 1) at the 4-position of thiazole ring. On this basis, the structure is modified to achieve the goal of additional diversity by linking piperidine at 1-position, replacing piperidine with piperazine and various alkyl groups (Compound 2), or replacing pentaaryl group in previous patents with 2,6-disubstituted pyridine (Compound 3). The publisher has declared that the IC50 value of the above series of compounds is in the range of 1–1,000 nM, but the Ki value has not been described. Therefore, it is difficult to compare the potency of the target compound without specific IC50 and Ki values. Unfortunately, it is unclear whether the action of such compounds is competitive with ATP or Sph, or non-competitive, nor is the use of these SphK1 inhibitors mentioned. But it is exciting that the patent mainly states the treatment of these SphK1 inhibitors for cancer and RA, which makes people look forward to the research on mechanism and pharmacological activity in the near future (Lynch et al., 2016).

### Pyrrolidine Analogue

#### PF-543

PF-543, developed by high throughput screening and medicinal chemistry optimization, is the most effective selective SphK1 inhibitor described so far (Figure 3J). Its selectivity is 100 times higher than that of SphK2 (Ki = 4.3 nM) discovered and reported by Pfizer (Schnute et al., 2012). Its chemical name is (2R)-1-[[4-[[3-Methyl-5-[(phenylsulfonyl)methyl]phenoxy]methyl]phenyl]methyl]-2-pyrrolidinemethanol hydrochloride, belonging to pyrrolidine analogues. Structurally, the basic skeleton of the compound is composed of a tertiary amine which form part of pyrrolidine ring, and the rest maintains the same 1,2-amino alcohol motif as Sph. A recent study shows that pyrrolidine inhibitors, especially with 2-hydroxymethylpyrrolidine structure, may be the key to the inhibitory potency (Li et al., 2021). Subsequently, the crystal structure of a hSphK1 binding to PF-543 was reported by Wang et al. (2014b). It was clarified that the inhibitor was bound in the SphK1 substrate pocket with a J-shaped structure. Specifically, the terminal phenylsulphonyl ring occupied the hydrophobic pocket formed by residues, including Phe374 and Leu347, 354 and 405. The (R)-2- (hydroxymethyl) - pyrrolidine head group is rotated to match the lipid primary hydroxyl group for phosphorylation, in which the hydroxyl and pyrrolidine nitrogen forms hydrogen bonds with the side-chain of Asp264. Finally, the central aromatic ring and its substituted methyl groups interact with Phe and Leu respectively. However, toluene group did not seem to be an essential group, which has no significant effect on SphK1 inhibition and anticancer activity (Kim et al., 2020). Compared with SphK2, SphK1 has three different residues in lipid binding sites, of which Phe374 and terminal phenyl ring may be the most closely bound part. The substitution of Cys in SphK2 by Phe374 may be an important reason for the high selectivity to SphK1 rather than SphK2.

PF-543 induced protein degradation of SphK1, which reduced the level of S1P and increased Sph. However, PF-543 had no effect on Cer level, suggesting that the lack of the ability to induce apoptosis (Byun et al., 2013; Wang et al., 2013). A recent study has proved that PF-543 reduces apoptosis in the lungs of mice after acute ethanol intoxication, and inhibits neutrophil infiltration and the release of inflammatory cytokines to reduce lung injury (Chen et al., 2021b). As the most effective SphK1 inhibitor so far, PF-543 can inhibit inflammation in RA model (Deng et al., 2021), ulcerative colitis model (Liu and Jiang, 2020) and mouse pulmonary hypertension hypoxia model (Ha et al., 2020) *in vivo* and *in vitro*, mainly by inhibiting the release of inflammatory cytokines and the change of cell biological function. In addition to inflammatory response, PF-543 plays a therapeutic role in angiogenesis in the pathogenesis of RA and microvascular leakage induced by sepsis (Zhong et al., 2020). Although these evidences show the beneficial effects of PF-543 on the pharmacological inhibition of SphK1, it is worth noting that the administration concentration of PF-543 needs to be further confirmed to ensure the specificity of SphK1.

#### CHJ01

A recent study found that a SphK1 selective inhibitor CHJ01 showed a unique therapeutic effect on RA. CHJ01 is an analogue of jaspine B, which is a naturally occurring anhydrophytosphingosine derivative isolated from the Okinawan marine sponge Pachastrissa sp. and Jaspis sp (Figure 3K). It can reduce intracellular S1P level and increase Cer level by inhibiting SphK1 (Kuroda et al., 2002; Salma et al., 2009). The hydrochloride of CHJ01 obtained by structural optimization has a good inhibitory effect on SphK1, but almost no inhibitory effect on SphK2 with the IC50 of 8.89 µM. Structurally, the replacement of the furan ring by a pyrrole ring in CHJ01 may account for the stronger activity. Pharmacological experiments showed that CHJ01 exhibited anti-inflammatory effects similar to those of methotrexate *in vitro* and *in vivo* by reducing the swelling volume, arthritis score, spleen index and IL-1β, TNF-α, IL-6 levels in arthritis model rats, which contributed to the significant improvement of RA symptoms (Chen et al., 2021a).

#### Compound 51

A series of potent SphKs inhibitors based on an N-(5-alkyloxadiazol- 3-yl)benzyl)-3-hydroxypyrrolidine-2-carboxamide scaffold proposed by Genzyme, structurally similar to PF-543 but with better metabolic stability (Figure 3L) (Xiang et al., 2010). For example, the pyrrolidine group`of compound 17 was replaced by a hydroxyl group instead of methanol, in contrast to PF-543. Further optimization of compound 17 by Xiang et al. found that shortening of the straight alkyl chain leads to loss of inhibitor viability. In order to maintain activity, the attachment of a two-carbon alkyl linker between the oxadiazole ring and the carbocyclic ring increased activity as well as solubility. The resulting compound 51 is characterized by a cyclopentethyl group attached on the oxadiazole ring, chemical name (2S,3S)-N-((S)-1-(4-(5-(2-cyclopentylethyl)-1,2,4-oxadiazol-3-yl)phenyl)ethyl)-3-hydroxypyrroli-dine-2-carboxamide, showed excellent inhibitory effect on SphK1 with the IC50 of 0.058 μM. The pharmacokinetics study of compound 51 found the moderate oral bioavailability, qualified half-life in blood circulation, and good internal clearance, particularly for human liver microsomes (Table 1).

**TABLE 1:** SphK1 inhibitors and potential functions.

Classification	Inhibitors	Targeted molecules	Dosage	Effects	Potential mechanisms	Characteristic	References
SphK1 selective inhibitors (Sphingosine Analogues)	SK1-I (BML258)	SphK1	Ki = 10 μM	S1P levels↓, cell apoptosis↑, inflammation↓, autophagy↑	Increases the expression of ceramide derivatives, decreased S1P level, mediate ERK1/2 and Akt signal, increased the transcriptional activity of tumor suppressor protein TP53	No activity at PKCα, PKCδ, PKA, AKT1, ERK1, EGFR, CDK2, IKKβ or CamK2β; high solubility and delivery *in vivo*	Paugh et al. (2008); Pitman and Pitson (2010); Nagahashi et al. (2012); Lima et al. (2018); Chahar et al. (2021)
			0–20 μM				
			75 mg/kg				
	LCL351/146	SphK1	Ki = 5 μM, 50 μM	S1P levels↓, pro-inflammatory cytokine↓, no effect on cell death and cell cycle, neutrophil infiltration and immune responses↓	induced SphK1 degradation	Two erythro diastereoisomers; a novel therapeutic target for IBD	Sharma (2011); Pulkoski-Gross et al. (2017)
	6ag/9ab/12aa	SphK1	IC50 = 0.65, 0.05, 0.062 μM	—	—	Significantly more potent than the previously reported SphK1 inhibitor N, N-dimethylsphingosine; 3-hydroxyproline improves the activity and ADME characteristics *in vitro*	Xiang et al. (2009)
(Amidine Inhibitors)	VPC96091	SphK1	Ki = 0.1, 1.5 μM	S1P levels↓	Increased Akt/ERK phosphorylation	An effective selective SphK1 inhibitor, reduced the S1P level of human leukemia U937 cells and mice	Foss et al. (2009); Kharel et al. (2011); University of Virginia Patent Foundation (2014); Kotthaus et al. (2011)
	SLP 7111228	SphK1	Ki = 48 nM	S1P levels↓	—	An effective selective inhibitor of SphK1; engagement of the target is indexed by blood S1P levels	Kharel et al. (2012); Patwardhan et al. (2015)
	Compound 28	SphK1	Ki = 0.3 μM, 6 μM	S1P levels↓	initiate growth arrest	No activity at DAG (γ, δ1, ζ), PKC α	Mathews et al. (2010)
(Piperidine Analogue)	Compound 82	SphK1	IC50 = 0.02, 0.1 μM	S1P levels↓, good mean residence time, and bioavailability of 32%	Hydrogen bonds are formed by the aminoalcohol portion of compound 82 and two key residues of aspartate in SphK1	Competitively inhibits SphK1; no activity at anti-tumor	Gustin et al. (2013); Wang et al. (2013); Rex et al. (2013)
	RB-005	SphK1	IC50 = 3.6 μM	The inhibition of ceramide synthase	Degradation of SphK1 proteasome↑	Derivative obtained from the route of synthesizing FTY-720; therapeutic potential for proliferative diseases, including PAH; potential inhibition of ceramide synthase	Baek et al. (2013a); Baek et al. (2013b); MacRitchie et al. (2016)
	Compound1/2/3	SphK1	IC50 = 1–1,000 nM	—	—	A series of selective inhibitors of SphK1; Therapeutic potential for RA and cancer	Frank (2013); Lynch et al. (2016)
(Pyrrolidine Analogue)	PF-543	SphK1	IC50 = 2 nM; Ki = 3.6 nM; 10–1,000 nM; 1 mg/kg	apoptosis↑, necrosis↑, and autophagy↑, S1P levels↓, Sph↑, no effect on the level of ceramide, inflammation↓	Degradation of SphK1 proteasome↑; inhibition of neutrophil infiltration and inflammatory cytokine release	A potent, selective, reversible and Sph-competitive SphK1 inhibitor; inhibitor was bound in the SphK1 substrate pocket with a J-shaped structure	Schnute et al. (2012); Byun et al. (2013); Wang et al. (2014b); Ha et al. (2020); Kim et al. (2020); Liu and Jiang (2020); Zhong et al. (2020); Chen et al. (2021b); Deng et al. (2021)
	CHJ01	SphK1	IC50 = 8.89 μM	S1P levels↓, Cer levels↑	Decrease of inflammatory cytokines in RA rat model	Effective treatment of RA *in vivo* and *in vitro*	Kuroda et al. (2002); Salma et al. (2009); Chen et al. (2021a)
	Compound 51	SphK1	IC50 = 0.058 μM	Modest oral bioavailability, qualified half-life in blood circulation	—	Better metabolic stability than PF-543	Xiang et al. (2010)
Dual SphK1/2 inhibitor (Non-Lipid Small Molecules)	SKI-I	SphK1, SphK2, ERK2, PKC, PI3K	IC50 = 1.2 μM	S1P levels↓, Cer levels↑, apoptosis↑, autophagy↑	—	a competitive Sph inhibitor of SphK1 and SphK2; anti-tumor activity	French et al. (2003); French et al. (2006); Hengst et al. (2010); Young et al. (2012)
	SKI-Ⅱ (SKi)	SphK1/2	Ki = 16, 8 μM	S1P levels↓, Ceride and Sph levels↑, apoptosis ↓, proliferation and migration↓, inflammation↓, oxidative stress↓	Triggering iysosomal degradation of SphK1; inhibit the activity of Des1; destroying the negative regulator Keap1	Oral bioavailable properties; the prevention of oxidative stress	French et al. (2003); Loveridge et al. (2010); Ren et al., 2010; Cingolani et al. (2014); Noack et al. (2014); Yang et al. (2015); Aurelio et al. (2016); Bien-Möller et al. (2016); McNaughton et al. (2016); Sun and Wang (2021)
	MP-A08	SphK1/2	Ki = 27, 7 μM	S1P levels↓, Cer and Sph levels↑, apoptotic↑, proliferation↓, tumor angiogenesis↓	No effects on proteasome degradation; target the ATP binding pocket of SphK1	An ATP competitive dual SphK1/2 inhibitor; higher affinity for SphK2; overcomes off-target effects	Cingolani et al. (2014); Pitman et al. (2015)
	DHS (Safingol)	SphK1, SphK2, PKC-α	Ki = 3–6 μM	Anticancer activity, apoptosis↑	—	A SphK1 competitive inhibitor; the first SphKs inhibitor to enter clinical trials as an anticancer agent	Schwartz et al. (1997); Coward et al. (2009); Dickson et al. (2011)
(Lipid Small Molecules)	DMS	SphK1, SphK2, PKC, CERK	Ki = 16 μM	Anticancer activity, apoptosis↑, hemolysis↑	—	The first direct SphKs inhibitor	Sweeney et al. (1996); Yatomi et al. (1996); Edsall et al. (1998); Sah et al. (2021)
Other SphK1 inhibitors	SKI-178	SphK1/2	Ki = 1.33 μM	apoptotic↑, cell death↑, anti-cancer↑	—	ATP non-competitively and selectively inhibits	Dick et al. (2015); Hengst et al. (2017); Hengst et al. (2020b)
	SK-F	SphK1	Ki = 1 μM 5 mg/kg	Cell growth↓, viability↓, anti-tumor↑	—	A selective and competitive SphK1 inhibitor; no significant systemic toxicity in the treatment of mouse breast tumors	Alshaker et al. (2018)
	SLC 4011540	SphK1/2	Ki = 120 nM, 90 nM	S1P levels↓, Sph remained unchanged	—	SphK1/2 dual inhibitor; characteristic feature is the presence of an electron-deficient phenyl ring	Childress et al. (2017)
	B-5354C/F-12509a	SphK1; SphK1/2	Ki = 18; 12 μM	S1P levels↓, Cer and Sph levels↑, cell death↓	—	Isolated by extraction from a discomycete, Trichopezizella barbata and a novel marine bacterium; non-competitive inhibitor	Bonhoure et al. (2006); Cuvillier (2008)
	11b	SphK1	IC50 = 3.1 μM	—	—	A hydrophobic moiety with naphthalene ring substituent; interaction with asp178	Gustin et al. (2013); Vettorazzi et al. (2020)
	Balanocar-pol	SphK1	Ki = 160 ± 40 μM	Cell apoptosis↑, proliferation↓	Changes in the SphK1 protein turnover	Sph-competitive inhibitor	Nakagawa et al. (2001); Sahidin et al. (2005); Loveridge et al. (2010); Lim et al. (2012)

## Dual SphK1/2 Inhibitor

### Non-Lipid Small Molecules

#### SKI-I

**FIGURE 4:**
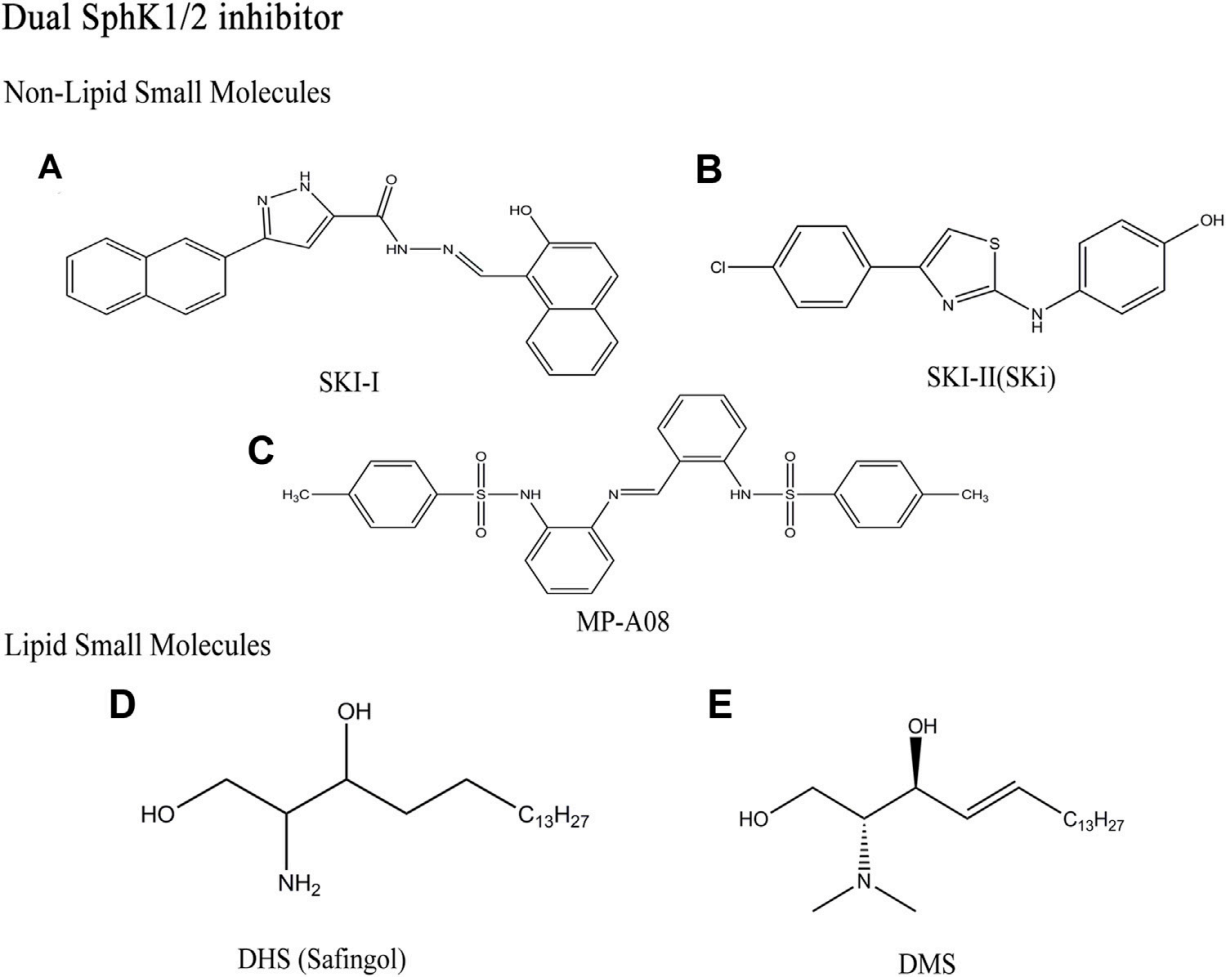
Structures of dual SphK1/2 inhibitors.

SKI-I, (N’-[(2-hydroxy-1-naphtyl)methylene]-3-(2-naphthyl)-1H-pyrazole-5-carbohydrazide), is also a competitive Sph inhibitor of SphK1, which was found by French et al. (Figure 4A) (French et al., 2003). SKI-I inhibit SphK1 competitively with IC50 of 1.2 μM, but it also inhibit SphK2 with similar affinity, and cross react with ERK2, PKC and PI3K (Hengst et al., 2010). Many *in vitro* and *in vivo* studies showed that SKI-I not only down-regulates S1P level and up-regulates Cer level, but also induces apoptosis and autophagy in T24 bladder cancer cells and mouse embryonic fibroblast (Young et al., 2012). In addition, the compound showed anti-tumor activity in mouse melanoma and breast cancer xenograft models (French et al., 2006).

#### SKI- Ⅱ(SKi)

SKI-Ⅱ, 2-(p-hydroxyanilino)-4-(p-chlorophenyl)thiazole, was identified from a high throughput screening by French et al. (Figure 4B) (French et al., 2003). As non-lipid small molecule compound with dual SphK1/2 inhibitors, SKI-Ⅱ showed stronger inhibitory effect of SphK1 than SphK2 (Ki (SphK1) = 16 μM, Ki(SphK2) = 8 μM). Similar to SKI-I, SKI-Ⅱ attenuates SphK1 signaling by triggering lysosomal degradation of SphK1 in different cell types, mainly by binding to an allosteric site, which is different from the enzyme sites promoting polyubiquitination and proteasome degradation of SphK1 (Loveridge et al., 2010; Ren et al., 2010). SKI-Ⅱ has no inhibitory effect on ERK2, PKC and PI3K compared with SKI-I. SKI-Ⅱ induces apoptosis and inhibits proliferation and migration by increasing Cer/Sph and reducing S1P levels (Bien-Möller et al., 2016; Sun and Wang, 2021). The effect of reducing inflammation and acute myeloid leukemia (AML) *in vivo* has been reported due to oral bioavailability of SKI-Ⅱ (Yang et al., 2015). Recent studies have shown that SKI-Ⅱ inhibit the activity of dihydroceramide desaturase-1 (Des1) with Ki of 0.3 μM, which is related to the prevention of oxidative stress (Cingolani et al., 2014; Noack et al., 2014). SKI-Ⅱ stabilize the redox sensitive transcription factor nuclear factor-erythroid-2-related factor 2 (Nrf2) and play a protective role in diseases caused by oxidative stress by destroying the negative regulator Keap1 (McNaughton et al., 2016). Aurelio et al. further proved that the anti-proliferative effect of SKI-Ⅱ and its analogues is mainly due to Des1 inhibition rather than the binding of allosteric sites (Aurelio et al., 2016).

#### MP-A08

MP-A08, chemical name 4-methyl-N-[2-[[2-[(4-methylphenyl)sulfonylamino]phenyl]iminomethyl]phenyl]benzenesulfona-mide, found by homology modeling of the ATP binding site of SphK1 (Figure 4C) (Pitman et al., 2015). MP-A08 is an ATP competitive dual SphK1/2 inhibitor with corresponding Ki values of 27 and 7 μM. This indicates that MP-A08 targets both SphK1 and SphK2, but has higher affinity for SphK2. Some SphKs inhibitors, such as SKI-Ⅱ and PF-543, have been found to inhibit SphK1 in cells by targeting proteasome degradation, which seems to be a feature of many Sph competitive SphKs inhibitors. However, MP-A08 had no effect on SphK1 degradation, which further proved that the inhibitor had higher affinity with SphK2. On the other hand, early SphK1 inhibitors mostly acted against the Sph binding pocket, which is highly likely to have off-target effects due to the molecular structure retaining Sph characteristics (Cingolani et al., 2014). Whereas the selectivity for SphK1 and SphK2 is higher than other kinases, because MP-A08 targeting the ATP-binding pocket is structurally different from protein kinases and almost all other lipid kinases. In brief, MP-A08, an inhibitor developed to target the ATP-binding pocket of SphK1, which not only takes advantage of the known divergence of the SphK1 ATP-binding site from other protein kinases to improves selectivity, but also overcomes off-target effects common to molecules like Sph. MP-A08 increased Sph and Cer levels and decreased S1P levels, shifting tumor cells from an anti-apoptotic, pro-proliferative to a pro-apoptotic and anti-proliferative phenotype and blocking the survival of multiple cancer cell lines (Pitman et al., 2015).

### Lipid Small Molecules

#### DHS (Safingol)

The D, L-threo-dihydrosphingosine (DHS), a SphK1 competitive inhibitor with Ki of 3–6 μM, is the first SphK1 inhibitor reported in the literature (Figure 4D) (Coward et al., 2009). Later studies have shown that this compound can also participate in sphingolipid metabolism pathway as SphK2 substrate, and inhibit other kinases (such as PKC-α), to produce other non-target effects. DHS, also known as Safingol, has a large number of experimental researches before clinical trials. It enhances the anti-tumor effect of various chemotherapeutic drugs (such as doxorubicin) *in vitro* by inducing apoptosis (Schwartz et al., 1997). As the first SphKs inhibitor to enter clinical trials as an anticancer agent, it has obvious anticancer activity *in vitro* and can be safely administered in combination with cisplatin (Dickson et al., 2011).

#### DMS

DMS, N,N-dimethyl-D-erythro-sphingosine, is the first direct SphKs inhibitor with Ki of 16 μM at SphK1, which is similar to DHS (Figure 4E) (Yatomi et al., 1996). It blocks the activity of the two isozymes by competing with the natural substrate Sph. DMS inhibit the growth of tumor, induce apoptosis of cancer cells, and block PKC signaling (Edsall et al., 1998). In addition, DMS has been shown to inhibit SphK2 and ceramide kinase (CerK), which makes it unable to act on SphK1 specifically. Although the inhibitors have been found to have therapeutic effects on many types of cancer cells and tumors *in vitro* and *in vivo*, but severe hemolysis in mice is triggered (Sweeney et al., 1996; Sah et al., 2021). Obviously, the non-targeting effect of sphingolipid analogue inhibitors makes it unable to be an ideal SphKs inhibitor (Table 1).

## Other Sphingosine Kinase 1 Inhibitors

### SKI-178

**FIGURE 5:**
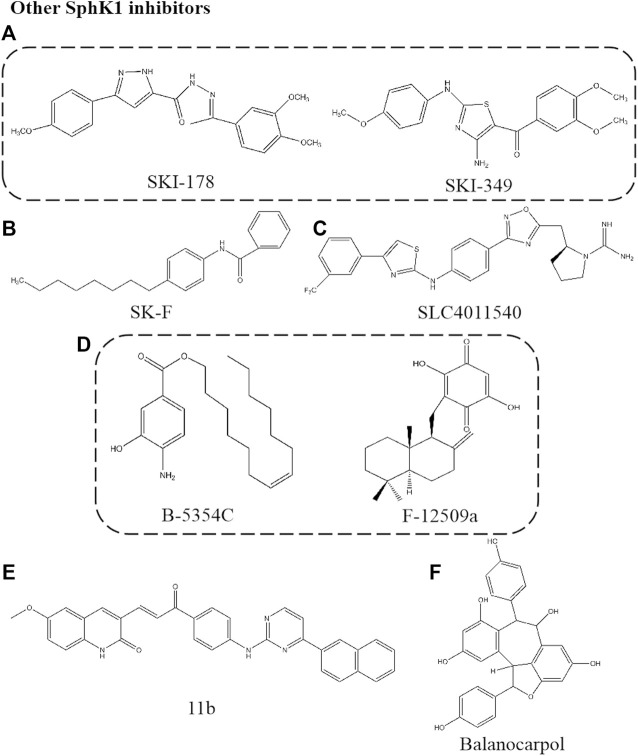
Structures of other SphK1 inhibitors.

SKI-178, N’-[1-(3,4-dimethoxyphenyl) ethylidene]-3-(4-methoxyphenyl)-1H-pyrazole-5-carbohydazide, is an analogue of SKI-I (Figure 5A). SKI-178, as a non-lipid small molecule SphK1 selective inhibitor, binds to ATP non-competitively and inhibits the activity of SphK1 selectively, with Ki of 1.33 μM. This compound inhibits the role of SphK1 in tumorigenesis and progression *in vivo* and *in vitro* due to the high selectivity and low toxicity. It was found that SKI-178 also target SphK2 to induce apoptosis of AML cell line and microtubule destruction by cell thermal shift assay (CETSA) analysis (Dick et al., 2015; Hengst et al., 2017). This team modified the “linker region” between the substituted phenyl rings of SKI-178 to obtain SKI-349, which showed a logarithmic improvement in SphKs inhibitory potency and therapeutic efficacy on AML model (Hengst et al., 2020b).

### SK-F

SK-F is a selective SphK1 inhibitor obtained by structural analysis of three known SphK1 inhibitors, SKI-178, 12aa and SK1-I (Figure 5B). Electrostatic and van der Waals field points for each conformation of the three SphK1 inhibitors were calculated, and features of these molecular field patterns common to them were extracted in the form of field templates, searching for patterns related to polar headgroups, and designing new compounds. The resulting new compound SK-F was shown to be a potent SphK1 inhibitor by SphK1 activity assays *in vitro*. The compound SK-F effectively reduced cancer cell viability *in vitro* and sensitized mouse mammary tumors to docetaxel *in vivo* without significant systemic toxicity (Alshaker et al., 2018).

### SLC4011540

This is a SphKs inhibitor of guanidine compounds containing aminothiazole (Figure 5C). The skeleton of the compound is an oxadiazole phenyl ring with an aminothiazole structure and a guanidine scaffold as head group. SLC4011540, a dual SphK1/2 inhibitor, Ki of 120 nM for SphK1 and Ki of 90 nM for SphK2. This class of dual SphK1/2 inhibitors are characterized by the presence of an electron-deficient phenyl ring, and this substitution may establish interactions with residues Cys533, His556, and Tyr566 at the end of the binding pocket. In addition, the exocyclic NH of the aminothiazole ring interacted with the target via hydrogen bonding. SLC4011540 effectively reduced the S1P level of U937 cells (a lymphoma bone marrow cell line expressing two SphKs isoforms) and Sph remained unchanged, indicating that this compound has cell permeability and effectively inhibits SphKs activity in cells (Childress et al., 2017).

### B-5354C/F-12509a

The non-Sph analogues F-12509a and B-5354C were isolated by extraction from a discomycete, Trichopezizella barbata and a novel marine bacterium, respectively (Figure 5D). The structure of F-12509a is a sesquiterpene quinone consisting of a drimane moiety and a dihydroxybenzoquinone. Similar to DMS, F-12509a competitively inhibited SphK1 with Ki of 18 μM, suggesting that the sesquiterpene moiety of F-12509a can mimic a Sph conformation when bound to the active site of SphK1. F-12509a increased Cer accumulation and decreased S1P level by inhibited SphK1 (Bonhoure et al., 2006). On the other hand, the structure of B-5354C is a ester of 4-amino-3-hydroxybenzoic acid with a longchain unsaturated alcohol with Ki of 12 μM. The difference is that B-5354C shows non-competitive inhibition, interacting with domains different from Sph related sites to regulate SphK1 activity (Cuvillier, 2008).

### 11b

The compound is a series of compounds with SphKs inhibitory activity synthesized by SKI- Ⅱ as the starting structure and designed by molecular modeling based on PF-543 (Figure 5E) (Vettorazzi et al., 2020). Firstly, the structure containing quinoline pharmacophore was designed based on SKI-Ⅱ. Unfortunately, the naphthyl residue located in the hydrophobic part did not enhance the efficacy, according to Gustin et al. (2013). Next, a new linker between the polar head and the hydrophobic tail was found to extend the structure, which can better combine with SphK1 crystal to improve the affinity. 11b shows selective inhibition on SphK1 with IC50 of 3.1 μM. We can find that the hydrophobic part of the molecule has the substituent of naphthalene ring, and the interaction with Asp178 (important residues anchored by ligands in SphKs active site) may be more advantageous for the inhibitory effect by comparing the structure. This structure and potential function has not been verified at the cellular level. However, the development of new inhibitors, such as the location and potential role of functional groups, can provide new strategies for the screening and structural optimization of inhibitors.

### Balanocarpol

Balanocarpol, as a dimer of resveratrol, was extracted and isolated from dried leaves of H. *dryobalanoides* (Figure 5F) (Lim et al., 2012). Balanocarpol was found to act as an inhibitor of SphK1 due to the inhibition of SphK1 activity and down-regulation of expression. Balanocarpol is a Sph competitive inhibitor of SphK1 with Ki of 160 ± 40 μM, although this represents a relatively low potency. Pharmacological studies found this concentration to be consistent with that inducing cancer cell apoptosis (Nakagawa et al., 2001). Balanocarpol induced down-regulation of SphK1 activity and expression in prostate cancer cells, the mechanism may involve changes in the SphK1 protein turnover, as has been shown for other SphK1 inhibitors to induce ubiquitin proteasomal degradation of SphK1 or changes in gene promoter activity (Loveridge et al., 2010). In addition, studies proved that balanocarpol may bind to only one catalytic site in the SphK1 dimer and exert inhibitory effects. The resveratrol tetramer, di-balanocarpol, inhibited cell proliferation potently more than balanocarpol, which supports the statement that the inhibitory potency of balanocarpol is enhanced by dimerization (Sahidin et al., 2005) (Table 1).

## Discussion

S1P is the first sphingolipid metabolite to attract attention due to its involvement in many important cellular processes as a bioactive mediator. S1P exerts different or even completely opposite roles through five specific GPCRs. There has been interest in SphKs as the only way to catalyze the generation of S1P, acting like a housekeeper to maintain sphingolipid-rheostat homeostasis to determine cell fate. The role of SphK1 is more clear than SphK2 with the development of research. SphK1 is abnormally activated (or inhibited) in the disease state. SphK1, which is no longer expressed stably, cannot maintain the balance of sphingolipid-rheostat, and the normal physiological level and homeostasis of internal environment are broken. It was found that SphK1 expression and activity are mostly up-regulated in a variety of pathological conditions, and SphK1 and SphK1/S1P signal is mainly involved in regulating inflammatory responses and inflammatory mediators. SphK1 mediates the secretion of inflammatory cytokines through related pathways, such as TNF family, ILs, chemokines, and adhesion molecules. It is involved in the regulation of various immune cell functions, such as proliferation, migration, activation, and intercellular interactions to regulate inflammatory response. Therefore, SphK1 is up-regulated in inflammatory immune related-diseases, including hypertension, atherosclerosis, AD, RA and various types of cancers, and becomes a new target for the treatment of diseases.

**FIGURE 6:**
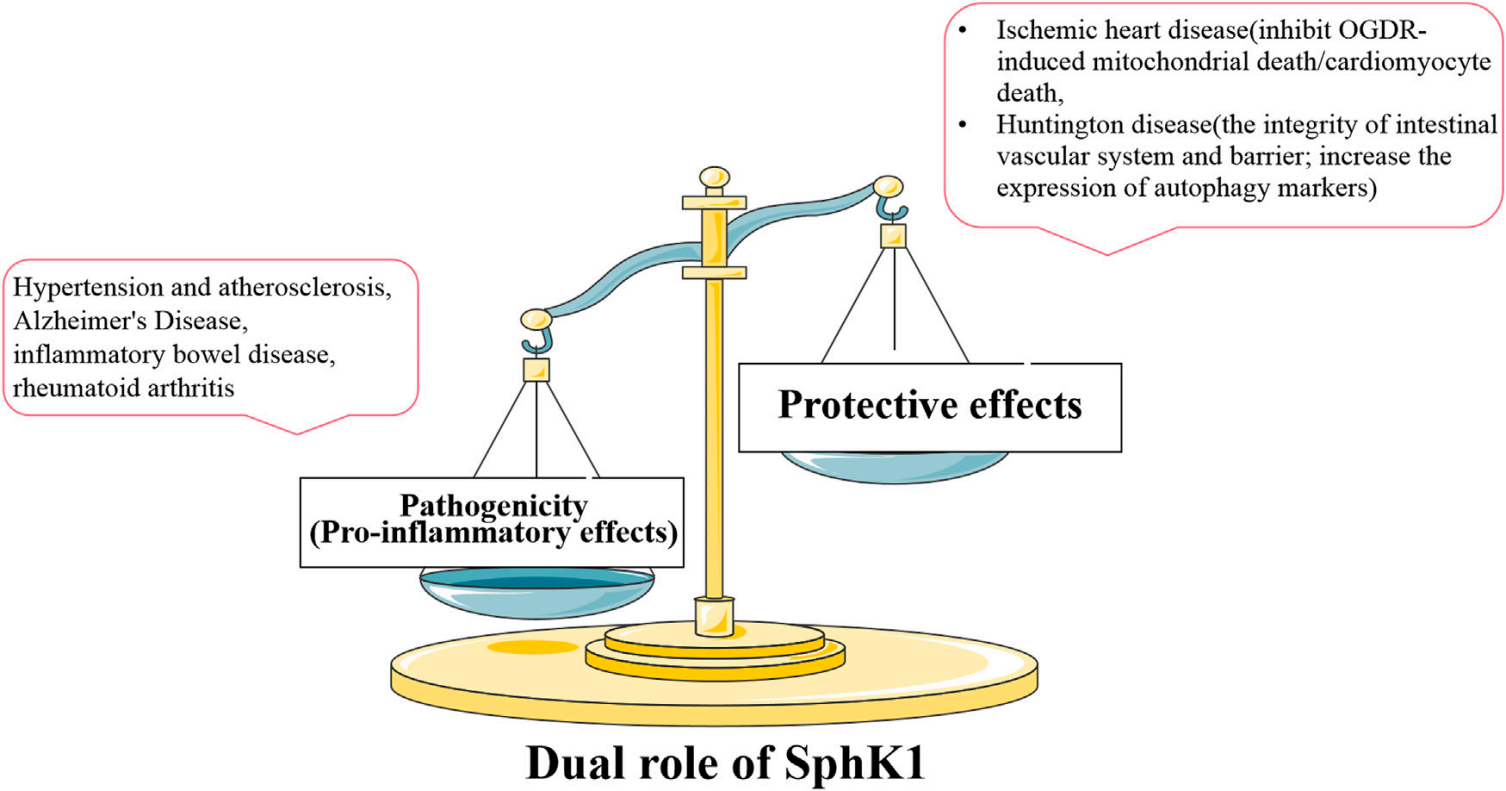
Relationship between the expression and activity of SphK1 and dual role in disease. SphK1 plays different roles in different diseases. In most inflammatory diseases, the increase of pathogenic SphK1 expression intensifies the development of the disease, and SphK1 inhibitors play a therapeutic effects. The decreased expression or activity of SphK1, which has a protective effect in other diseases, contributes to the development of the other diseases (such as ischemic heart disease and HD). Therefore, normal SphK1 level is very important for the stability of body function.

Interestingly, SphK1 also plays a protective role in some diseases different from the pathogenic role mentioned above. In Oxygen–Glucose Deprivation/Reoxygenation (OGDR)-induced cardiomyocyte injury, SphK1 agonist K6PC-5 increased intracellular S1P level and significantly inhibited OGDR-induced cardiomyocyte death. Inhibition of SphK1, whether pharmacological inhibition or SphK1 siRNA knockdown, aggravates cytotoxicity and invalidates the protective effect of K6PC-5. Mechanism studies showed that over-expression of SphK1 inhibited mitochondrial death induced by OGDR, including the production of ROS and the decrease of mitochondrial membrane potential (Shao et al., 2015). These results indicate that the over-expression of SphK1 can inhibit cardiomyocyte death and protect ischemic heart disease. It was also found that the up-regulated SphK1 could protect neurons from the effects of OGDR by inhibiting the programmed necrosis of neurons (Liu et al., 2018). The death of both cardiomyocytes and neurons is induced by the destruction of mitochondrial function. We reasonably speculate that SphK1 may play an important protective role in OGDR-induced mitochondrial death pathway. In addition, the protective effect of SphK1 was also found in Huntington disease (HD), a neurodegenerative disease with peripheral complications such as disturbance of gastrointestinal homeostasis. SphK1 expression was significantly reduced in the small intestine of R6/2 mice (a transgenic animal model of HD), which was consistent with observation in brain tissue (Di Pardo et al., 2017; Di Pardo et al., 2019). The up-regulation of SphK1 expression or treatment with K6PC-5 can maintain the integrity of intestinal vascular system, increase the expression of autophagy markers and alleviate progressive motion defects related to disease progression through *in vivo* and *in vitro* research (Di Pardo et al., 2020). The molecular mechanism of the protective effect of K6PC-5 on HD mice may depend on the correlation between SphK1 and autophagic flux. Although the above focuses on the over-expressed SphK1 in the disease state and the therapeutic effect of SphK1 inhibitors, we cannot ignore the protective effect of SphK1 in some diseases based on the therapeutic effect of K6PC-5 on ischemic heart disease and HD. Specifically, pathogenic SphK1 is involved in the pathogenesis of most inflammatory diseases, showing an increase in SphK1 expression or activity. SphK1 inhibitors reduce SphK1 expression or activity and play a therapeutic role in this case. However, the up-regulated SphK1 improves the other diseases (such as ischemic heart disease and HD), which is characterized by the decrease of SphK1 expression or activity in the disease state. This completely opposite effect is defined as the protective effect of SphK1. SphK1 agonists up-regulate the expression or activity of SphK1 and play a therapeutic role, while inhibitors aggravate the disease (Figure 6).

Whether it is pathogenic or protective, drugs targeting SphK1 aim to restore the balance of SphK1 expression or activity, which is consistent with the concept of “Soft regulation of inflammatory immune responses (SRIIR)” in immune inflammatory response (Wei, 2016). Inflammatory immune response is a physiological or excessive systemic response caused by inflammatory immune cells based on the changes of internal and external environment. The significance of SRIIR is to control the excessive activity of inflammatory immune related cells, restore the balance of inflammatory cytokines, and improve inflammatory diseases without damaging normal physiological function. At present, anti-inflammatory and immune drugs in clinical treatment show good therapeutic effects on inflammation related diseases, such as non-steroidal anti-inflammatory drugs, glucocorticoids, immunosuppressants and botanical drugs, but will have adverse side effects on the body. The goal of SRIIR drugs is to selectively regulate the balance of specific protein expression or activity, minimize adverse side effects, restore normal cell function and achieve homeostasis.

Although SphK1 and SphK2 have overall homology and common product S1P, some studies have found that SphK2 seems to show different functions from SphK1. For example, SphK1 and SphK2 have completely opposite regulatory effects in the inflammatory arthritis. SphK1 siRNA treatment significantly reduced the severity of the disease, while SphK2 siRNA treatment had no effect on the disease. However, ABC294640 (SphK2 selective inhibitor) leads to more severe arthritis (Lai et al., 2009; Xu et al., 2014). For SphK2, the difference in inflammatory phenotype between genetic inhibition and pharmacological inhibition is probably due to the difference between the rapid acute inhibitory effect induced by the dosage of inhibitors and the lifetime lack of genetic induction. SphK1 and SphK2 have redundant functions to compensate each other to achieve basic functions. Therefore, more rigorous evidence is needed for the development and research of selective inhibitors to avoid off-target effect and prove target specificity. This urgent need is due to the failure of iniparib in phase Ⅲ clinical, a PARP inhibitor as an anticancer drug, where iniparib was shown not to inhibit PARP activity but rather to non-selectively modify cysteine residues (Liu et al., 2012). Some studies have verified the target of existing SphK1 inhibitors and found that they also have affinity for another isotype (SphK2) at high concentration (micromolar level) (Hengst et al., 2020a). Therefore, the concentration of inhibitors should be carefully selected and combined with molecular tools to improve the preciseness and repeatability of the study when involves the differentiation of two enzyme isomers or the study of the biological function of one isomer.

In conclusion, SphK1 mediates a variety of functions of inflammatory cells, regulates the secretion of inflammatory factors, participates in the process of related diseases, and plays an important role in inflammatory and immune related-diseases. SphK1 inhibitors have been shown to improve cell biological function and disease symptoms by mediating inflammation and immune response. Therefore, SphK1 is becoming a new target for the development of relevant innovative drugs as a key regulator for the treatment of inflammatory and immune related-diseases.
